# Is Follow-up BRAF^V600E^ Mutation Analysis Helpful in the Differential Diagnosis of Thyroid Nodules with Negative Results on Initial Analysis?

**DOI:** 10.1371/journal.pone.0058592

**Published:** 2013-03-07

**Authors:** Jung Hyun Yoon, Eun-Kyung Kim, Hee Jung Moon, Jin Young Kwak

**Affiliations:** 1 Department of Radiology, Research Institute of Radiological Science, Yonsei University, College of Medicine, Seoul, Korea; 2 Department of Radiology, CHA Bundang Medical Center, CHA University, School of Medicine, Seongnam, Korea; Sapporo Medical University, Japan

## Abstract

**Background:**

We evaluated the usefulness of follow-up BRAF^V600E^ mutation analysis using ultrasonography-guided fine-needle aspiration (US-FNA) in diagnosis of thyroid nodules showing negative BRAFV600E mutation on prior analysis.

**Methodology/Principal Finding:**

A total of 49 patients (men: 6, women: 43, mean age: 50.4 years) with 49 thyroid nodules were included. Patients had undergone initial and follow-up US-FNA and subsequent BRAF^V600E^ mutation analysis from US-FNA aspirates. All patients had negative results on initial BRAF^V600E^ mutation analysis. Clinicopathologic findings, US assessment, and BRAF^V600E^ mutation results were analyzed according to the final pathology. Of the 49 nodules, 12 (24.5%) were malignant and 37 (75.5%) were benign. Seven (58.3%) of the 12 malignant nodules were positive for BRAF^V600E^ mutation on follow-up, all showing suspicious US features. Initial US-FNA cytology of the 7 nodules were non-diagnostic (n = 2), benign (n = 2), or atypia (n = 3), while follow-up were benign (n = 1), indeterminate (n = 1), suspicious for malignancy (n = 4), and malignancy (n = 1).

**Conclusions/Significance:**

Follow-up BRAF^V600E^ mutation analysis may be helpful in the diagnosis of selected thyroid nodules negative for BRAF^V600E^ mutation on initial analysis, which are assessed as suspicious malignant on US, diagnosed as non-diagnostic, benign or atypia on follow-up US-FNA.

## Introduction

Recent reports worldwide show rapid increase in the incidence of papillary thyroid carcinoma (PTC), the most common form of thyroid cancer [1]–[4]. Molecular genetics have evolved remarkably in the recent decade, and at the present time BRAF mutation is the most commonly known genetic alteration in PTC [5]–[6]. BRAF^V600E^ mutations found in PTC are a thymine to adenine transversion at nucleotide 1799 (T1799A), leading to a substitution of valine to glutamic acid at residue 600 of the protein (V600E)[5], [7]–[8]. This point mutation leads stimulation of MAPK pathway, which is results in oncogenic activation in the thyroid. BRAF^V600E^ mutation is common and highly specific for conventional PTC, and can be used for diagnosis, optimal sugical planning, and postoperative patient management [9].

Up to the present date, ultrasonography-guided fine-needle aspiration (US-FNA) has showed excellent performances and considered the standard diagnostic method in the diagnosis of various thyroid nodules detected on ultrasonography (US) [10]–[14]. Major limitations of US-FNA are the indeterminate cytology results such as ‘inadequate or non-diagnostic’, ‘follicular cells with atypia’, ‘follicular neoplasm’, or ‘suspicious for malignancy’, which consist of 10–30% of all cytology results [15]. BRAF^V600E^ mutation analysis has proven to be both useful and accurate as an adjunctive diagnostic tool to US-FNA in providing additional information in the differential diagnosis of PTC showing non-diagnostic or indeterminate cytology results [7], [16]–[18], especially in a BRAF^V600E^ mutation prevalent area such as Korea. Mostly, BRAF^V600E^ mutation analysis accompanies the initial US-FNA of a thyroid nodule based on clinical or radiological suspicion for malignancy, and has been proven more effective when performed at the time of initial cytologic analysis [17]. To our knowledge, no other studies have reported results on the diagnostic significances of performing consecutive follow-up BRAF^V600E^ mutation analysis on the differential diagnosis of thyroid nodules, especially in nodules with negative results on prior BRAF^V600E^ mutation analysis which had ambiguous cytology such as non-diagnostic and atypia, or discordant cytology to US features, and whether if this additional analysis of BRAF^V600E^ mutation is useful or not has not been established. Therefore, we evaluated the usefulness of follow-up BRAF^V600E^ mutation analysis using US-FNA in the differential diagnosis of thyroid nodules which were initially negative for BRAF^V600E^ mutation on prior analysis, and in which circumstances this additional analysis can be effectively used.

## Materials and Methods

This study has been approved by the institutional review board (IRB) of Severance Hospital, Yonsei University (Seoul, Korea) and informed consent has been waived by the IRB committee since the study design was in a retrospective form. Informed consent for US-FNA and BRAF^V600E^ mutation analysis was obtained from all patients included in this study, prior to all procedures.

### Patients

**Figure 1:**
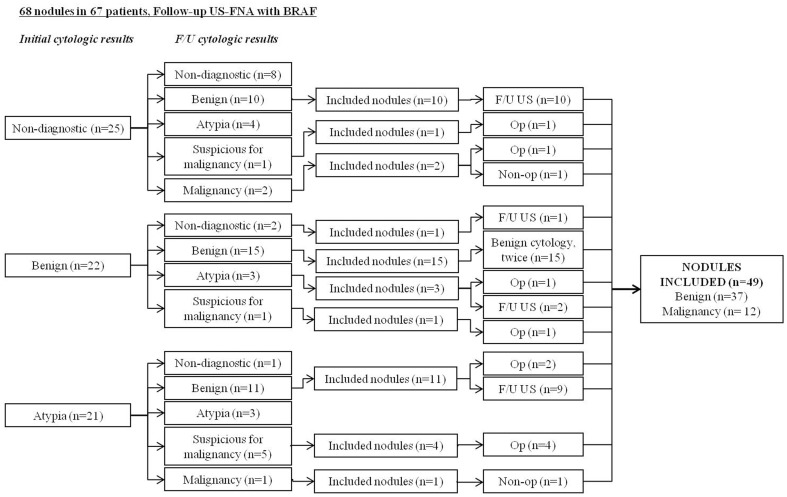
Inclusion of the study population. US-FNA: ultrasonography-guided fine-needle aspiration biopsy, F/U: follow-up, BRAF: BRAF^V600E^ mutation analysis, Op: operation.

This study was conducted at our institution (a referral center) from June 2009 to April 2011. During this period, 2,365 patients has undergone US-FNA and subsequent BRAF^V600E^ mutation analysis, of which 67 (2.8%) patients had undergone follow-up US-FNA and BRAF^V600E^ mutation analysis from the aspiration specimen in 68 thyroid nodules. Among them, nodules showing repeated non-diagnostic cytology on initial and follow-up US-FNA (n = 9), benign (n = 2), or atypia (n = 8) on initial US-FNA cytology without further follow-up surgical or imaging evaluation were excluded. Thyroid nodules with negative BRAF^V600E^ mutation results on initial US-FNA which fulfilled the following criteria were included: 1) surgery after US-FNA (n = 10), 2) benign cytology results on both initial and follow-up US-FNA (n = 15), 3) no changes on the follow-up US for at least 12 months after a benign cytology result on initial or follow-up US-FNA (n = 22), or 4) follow-up US-FNA cytology resulting in malignancy but without surgery (n = 2) [17]. A total of 49 thyroid nodules in 49 patients were finally included in this study (Fig 1).

At our institution, initial BRAF^V600E^ mutation analysis was performed based on requests from the referring clinicians based on clinical features of the patient, the judegements of radiologists performing US-FNA due to presence of suspicious US features of the targeted nodule during US examinations. Follow-up BRAF^V600E^ mutation analysis was recommended in patients with initial negative BRAF^V600E^ mutation by the clinician, due to clinial features arising suspicion for thyroid malignancy such as family history of PTC (n = 1) or prior non-diagnostic (n = 13), atypia of undetermined significance (n = 16) on initial US-FNA cytology and benign cytology results in spite of the presence of suspicious US features (n = 14), and unknown causes (n = 5).

### Imaging Analysis

US examinations and subsequent US-FNA procedures were performed using either 5– to 12– MHz linear probe (iU22, Philips Medical Systemts, Bothell, WA) or 6– to 13– MHz linear probe (EUB-7500, Hitachi Medical, Tokyo, Japan). Compoung imaging was performed when using the iU22 machine.

During the study period, US and US-FNA were performed by 1 of 14 board-certified radiologists with 1–14 years of experience in thyroid imaging. All radiologists had access to the medical record of the patients such as clinical symptoms, past history, and prior cytology and BRAF^V600E^ mutation analysis results when performing US and US-FNA. US features of the thyroid nodules were analyzed by the radiologist performing real-time US examinations prospectively, according to the following categories; internal composition, echogenicity, margin, presence of calcifications, shape and final assessment [19]. Malignant US features were defined as markedly hypoechogenicity (echogenicity lower than the adjacent strap muscle), non-circumscribed margins (microlobulated or irregular margins), microcalcifications (tiny, punctate, echogenic foci measuring less than 1 mm) [20] or mixed calcifications (mixed macrocalcifications, including eggshell calcifications, and microcalcifications), and non-parallel ‘taller-than-wide’ shape, based on a previously published criteria [19]. Final assessments were given as ‘probably benign’, when none of the suspicious US features mentioned above were present, and ‘suspicious malignant’, when 1 or more suspicious US features were present in the thyroid nodule. Final reporting and assessments of complicated cases was done after consulting senior faculty members.

### US-guided Fine-Needle Aspiration (US-FNA)

US-FNA was performed by the same radiologist who obtained real-time US images of the thyroid nodule. US-FNAs were performed on either thyroid nodules showing suspicious US features or the largest nodule with probably benign assessment on US, when none of the multiple thyroid nodules showed any suspicious US features.

US-FNA was performed at least twice from each targeted thyroid nodule using 23-gauge needles attatched to a 2 mL disposable plastic syringe, without an aspirator. Aspirated material was expelled on glass slides, which were smeared and immediately placed into 95% alcohol, for Papanicolaou staining. The remaining material in the syringe and needle hub was rinsed in normal saline for cell block processing. Cytopathologists were not present during US-FNA procedures, and additional staining of cytology slides were performed, if needed, on a case-by-case basis according to the request of the interpreting cytopathologist.

One of five cytopathologists specializing in thyroid pathology interpreted the slides obtained from US-FNA. During the study period of June 2009 to November 2009, cytology reports were divided into the following categories: 1) non-diagnostic, indicating specimen showing less than the required minimum of six groupings of well-preserved thyroid follicular cells, each consisting of at least 10 cells per group [21], 2) benign, 3) indeterminate cytology, which includes follicular neoplasm and Hürthle cell neoplasm (22–23). 4) suspicious for malignancy, and 5) malignancy. From December 2009 to the present, the Bethesda System for reporting thyroid cytopathology was used in the classification of cytology reports [21].

### BRAF^V600E^ Mutation Analysis

After US-FNA for cytology examinations, aspiration was performed once more for BRAF^V600E^ mutation analysis. Aspirated material obtained was rinsed in 1 mL of normal saline, which was sent for BRAF^V600E^ mutation analysis.

Direct DNA sequencing was used in mutation analysis. Exon 15, which contains the BRAF^V600E^ mutation, was amplified by PCR with the foward primer AGGAAAGCATCTCACCTCATC and the reverse primer GATCACACCTGCCTTAAATTGC. The PCR parameters were as follows: 94°C for 5 minutes, 35 cycles at 94°C for 0.5 minutes, 60°C for 0.5 minutes, and 72°C for 10 minutes. The amplified products were purified with a QIAGEN PCR purification kit and sequenced using the foward primer described previously with Big Dye Terminator (ABI Systems, Applied Biosystems, Foster City, CA), and an ABI PRISM 3100 Avant Genetic Analyzer (Perkin-Elmer).

### Data and Statistical Analysis

Histopathology results from surgery were considered the standard reference. Cytology results were used as standard reference for nodules which had not undergone surgery. Thyroid nodules showing benign cytology results on either initial or follow-up US-FNA cytology which did not show change on follow-up US for at least 1 year were considered benign. Nodules diagnosed as malignancy on US-FNA cytology which had not undergone surgery were considered malignant.

Statistical comparison was performed using *t*-test for parametric variables. *Χ* ^2^-test or Fisher's exact test was used in comparison of non-parametric variables. Generalized estimated equation (GEE) methods were used in comparing diagnostic performances. *P* values of less than 0.05 were considered to have statistical significance. Statistical analyses were performed using SAS software (SAS system for Windows, version 9.1.3; SAS Institute, Cary, NC).

## Results

Of the 49 thyroid nodules included in this study, 12 (24.5%) were malignancy and 37 (75.5%) were benign. Of the 49 patients, 6 (12.2%) were men and 43 (87.8%) were women. Mean age of the patients were 50.4 years (range, 29 to 67 years), and mean size of the thyroid nodules were 12.0 mm (range, 3 to 41 mm). Mean interval between the initial and follow-up US-FNA and BRAF^V600E^ mutation analysis was 6.3 months (range, 3 to 21 months).

Clinical, radiological, and cytopathologic features of the patients and thyroid nodules according to final pathology are summarized in Table 1. Mean size of the thyroid nodules were significantly smaller in malignancy (7.18±2.93 mm) compared to benign (13.43±8.76 mm, *P* = 0.023). No significant differences were seen in mean age and gender between the benign and malignant nodules.

**Table 1:** Clinical, radiological, and cytopathologic characteristics of the 49 patients and thyroid nodules according to final pathology.

	Benign	Malignancy	*P*
Number of nodules	37 (75.5%)	12 (24.5%)	-
Mean age (years±SD)	50.73±9.40	49.50±10.39	0.431
Mean size of nodules (mm±SD)	13.43±8.76	7.18±2.93	0.022
Patient gender			0.634
Men (%)	5 (13.5)	1 (8.3)	
Women (%)	32 (86.5)	11 (91.7)	
US Assessment			0.004
Probably benign (%)	17 (45.9)	0 (0.0)	
Suspicious malignant (%)	20 (54.1)	12 (100.0)	
2nd BRAF^V600E^ mutation			<0.001
Negative (%)	37 (100.0)	5 (41.7)	
Positive (%)	0 (0.0)	7 (58.3)	

SD: standard deviation

SD: standard deviation

On US examinations, all 12 (100.0%) malignant nodules showed 1 or more suspicious US features, while 20 (54.1%) benign ones were assessed as suspicious malignant on US final assessment. Malignant nodules showed significantly higher rates of suspicious US features compared to benign ones (*P* = 0.004). Seven (14.3%) of the 49 thyroid nodules was positive for the second BRAF^V600E^ mutation analysis, among which all were proven as malignancy (Table 2). Initial US-FNA cytology of the 7 nodules were non-diagnostic (n = 2), benign (n = 2), or atypia of undetermined significance (n = 3), while follow-up US-FNA cytology were benign (n = 1), indeterminate (n = 1), suspicious for malignancy (n = 4), and malignancy (n = 1).

**Table 2:** Demographics of the 7 patients with positive BRAF^V600E^ mutation on follow-up analysis.

No.	Age	Gender	Size (mm)	Inital US-FNA	Follow-up US-FNA	Final diagnosis
1	46	F	8	non-diagnostic	suspicious for malignancy	cPTC based on surgery
2	57	F	10	non-diagnostic	malignancy	Follow-up evaluation based on cytology
3	44	F	4	benign	indeterminate	cPTC based on surgery
4	45	F	7	benign	suspicious for malignancy	cPTC based on surgery
5	38	M	8	atypia	benign	cPTC based on surgery
6	54	F	5	atypia	suspicious for malignancy	cPTC based on surgery
7	58	F	6	atypia	suspicious for malignancy	FVPTC based on surgery

F: women, M: men

cPTC: conventional papillary thyroid carcinoma, FVPTC: follicular variant papillary thyroid carcinoma

Of the 17 nodules with probably benign US final assessment, initial US-FNA cytology results are as follows; 4 of non-diagnostic, 6 of benign, and 7 of atypia of undetermined significance. All 17 nodules were diagnosed as benign on follow-up cytology, showed negative results on follow-up BRAF^V600E^ mutation analysis, and did not show significant changes on the serial follow-up US examinations.

**Figure 2:**
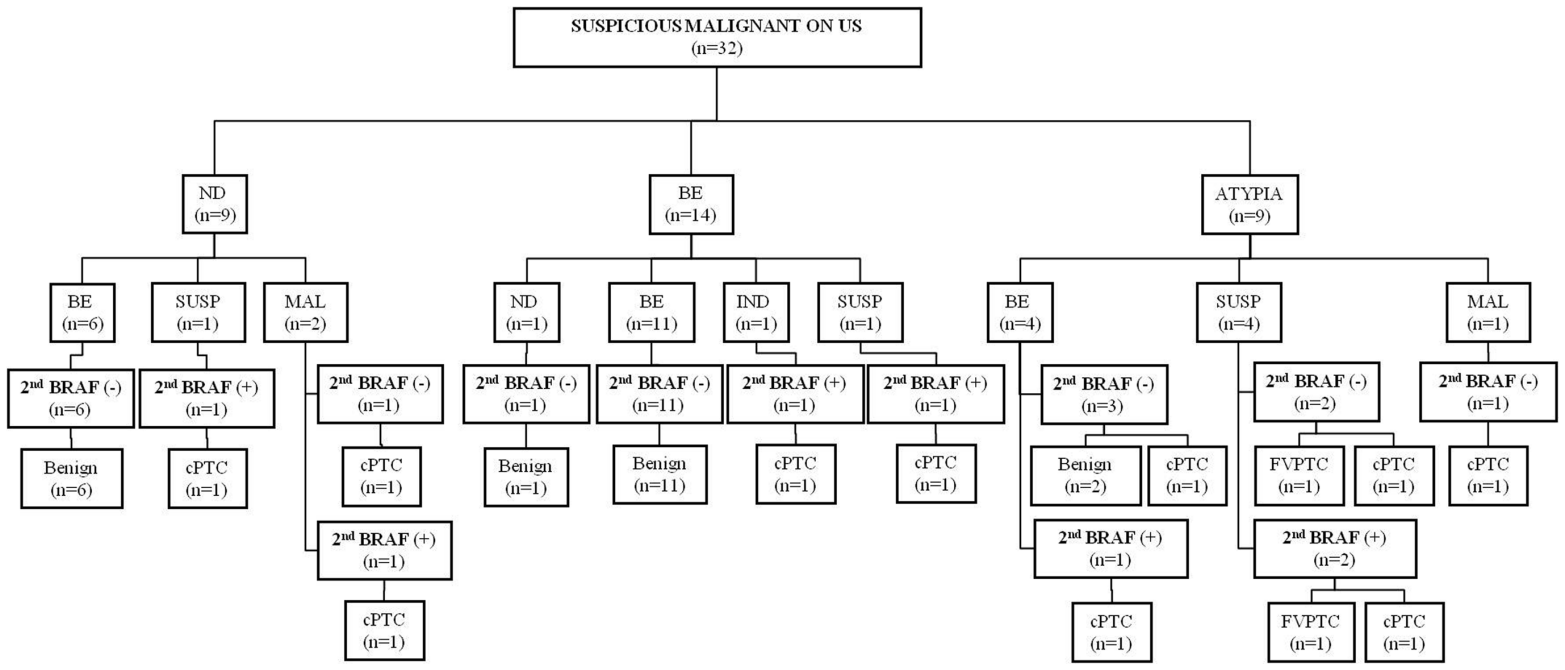
Final outcome of the 32 thyroid nodules with suspicious malignant US assessment. ND: non-diagnostic, BE: benign, IND: indeterminate, SUSP: suspicious for malignancy, MAL: malignancy, cPTC: conventional papillary thyroid carcinoma, FVPTC: follicular variant of papillary thyroid carcinoma

**Figure 3:**
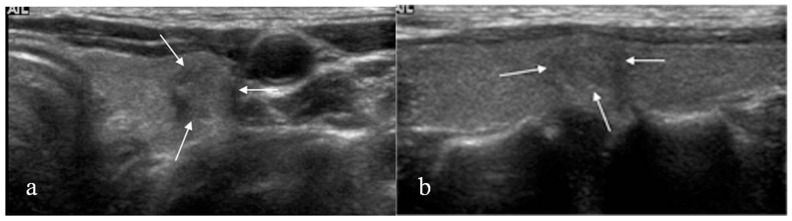
46-year-old woman with multiple thyroid nodules. US (a: transverse, b: longitudinal) revealed a 8-mm sized hypoechoic nodule (arrows) with peripheral calcifications in the mid pole of left thyroid. US assessment was suspicious malignant. Initial US-FNA showed non-diagnostic cytology and negative for BRAF^V600E^ mutation. This nodule was diagnosed as suspicious for malignancy on follow-up US-FNA performed 11 months later, and positive for BRAF^V600E^ mutation. Surgery confirmed this lesion as PTC.

**Figure 4:**
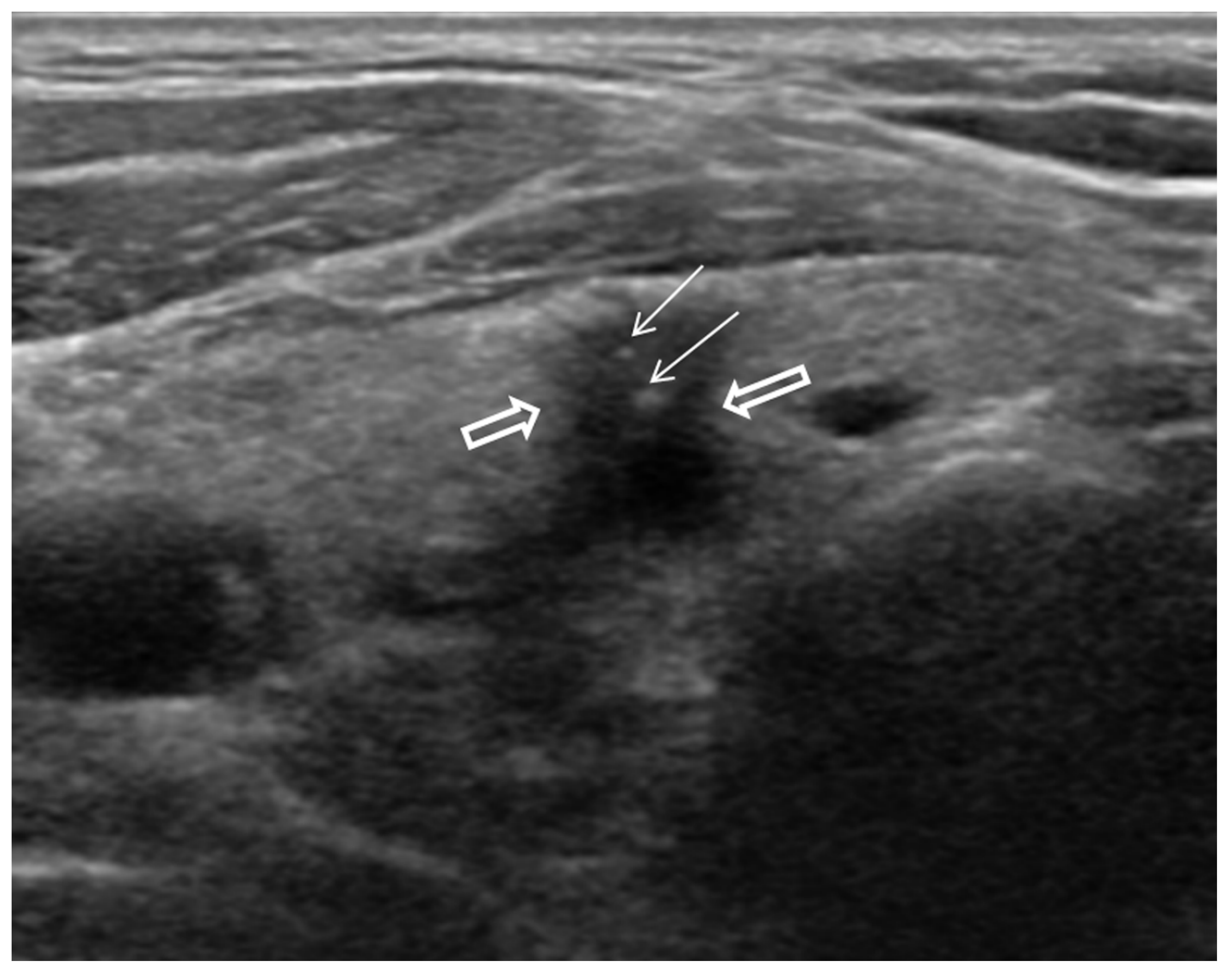
38-year-old man with multiple thyroid nodules. A 8-mm sized hypoechoic nodule (empty arrows) with suspicious calcifications (arrows), and spiculated margins was seen in right lower isthmus on US. US assessment was suspicious malignant. Initial US-FNA was diagnosed as atypia of undetermined significance, and negative for BRAF^V600E^ mutation. Follow-up US-FNA and BRAF^V600E^ mutation analysis was performed 6 months later, and follow-up cytology was diagnosed as benign, but positive results on BRAF^V600E^ mutation analysis. Surgery was performed, and this nodule was confirmed as PTC.

Final outcome of the 32 nodules with suspicious malignant US assessment are summarized in Figure 2. Of the 32 nodules with suspicious malignant US assessment, 12 (37.5%) were malignancy and 20 (62.5%) were benign on final pathology. Of the 9 nodules with initial non-diagnostic cytology, 3 nodules were diagnosed as suspicious for malignancy (n = 1) or malignancy (n = 2) on follow-up US-FNA. All 3 nodules were confirmed as PTC with surgery, among which 2 showed positive results on follow-up BRAF^V600E^ mutation analysis (Fig 3). Of the 14 nodules with initial benign cytology, 2 were diagnosed as indeterminate (n = 1) or suspicious for malignancy (n = 1) on follow-up US-FNA, both with positive results on follow-up BRAF^V600E^ mutation analysis, and confirmed as PTC on surgery. Eleven of the 14 nodules were diagnosed was once again diagnosed as benign on follow-up US-FNA, showed negative results on follow-up BRAF^V600E^ mutation analysis. All 11 nodules were confirmed as benign. Of the 9 nodules with atypia on initial cytology, 4 were diagnosed as benign on follow-up US-FNA, of which 1 nodule was positive for BRAF^V600E^ mutation on follow-up analysis, and was confirmed as PTC on surgery (Fig 4).

Diagnostic performances of the follow-up US-FNA, follow-up BRAF^V600E^ mutation analysis alone, and follow-up US-FNA combined with BRAF^V600E^ mutation analysis are summarized in Table 3. Sensitivity of US-FNA combined to BRAF^V600E^ mutation analysis was significantly higher than US-FNA alone (*P* = 0.02), while none of the other values showed significant differences.

**Table 3:** Diagnostic performances of follow-up US-FNA, BRAF^V600E^ mutation analysis, and follow-up US-FNA combined to BRAF^V600E^ mutation.

All nodules (n = 49)	Follow-up US-FNA	BRAF^V600E^	Follow-up US-FNA+BRAF^V600E^	P^*^	Nodules with suspicious US feature (n = 32)	Follow-up US-FNA	BRAF^V600E^	Follow-up US-FNA+BRAF^V600E^	P^*^
Sensitivity	83.3 (10/12)	58.3 (7/12)	91.7 (11/12)	0.021	Sensitivity	83.3 (10/12)	58.3 (7/12)	91.7 (11/12)	0.021
Specificity	100.0 (37/37)	100.0 (37/37)	100.0 (37/37)	-	Specificity	100.0 (20/20)	100.0 (20/20)	100.0 (20/20)	-
PPV	100.0 (10/10)	100.0 (7/7)	100.0 (11/11)	-	PPV	100.0 (10/10)	100.0 (7/7)	100.0 (11/11)	-
NPV	94.9 (37/39)	88.1 (37/42)	97.4 (37/38)	0.167	NPV	90.0 (20/22)	80.0 (20/25)	95.2 (20/21)	0.289
Accuracy	95.9 (47/49)	89.8 (44/49)	98.0 (48/49)	0.23	Accuracy	93.8 (30/32)	84.4 (27/32)	96.9 (31/32)	0.241

Note: Raw data are in parentheses

PPV: positive predictive value, NPV: negative predictive value, ^*^: *P* values when comparing US-FNA and US-FNA+BRAF^V60^

## Discussion

There is no doubt in that US-FNA is a safe, easy, and reliable diagnostic method in the differential diagnosis of variable thyroid nodules, but US-FNA has its limitations in that up to 10–30% of the cytology derived from it can show inconclusive results, such as insufficient material or indeterminate morphologic criteria of the cells [15], [21]. Molecular analysis of cytology specimen has developed and contributed in providing additional information in the differential diagnosis of thyroid nodules, with BRAF, RAS mutations, and RET/PTC rearrangements most popularly used. BRAF^V600E^ mutation, in particular, is present in 29–70% of papillary thyroid carcinoma [7]–[8], [18], reaching up to 84% in Korea, a BRAF^V600E^ mutation-prevalent area [4], [22]. Many studies have looked into the factors related to BRAF^V600E^ mutation, mostly focusing on clinical [9], [23]–[25], imaging or cytology features [1], [26]–[31]. However, to our knowledge there are no studies in published literature that have investigated the usefulness of follow-up BRAF^V600E^ mutation analysis among nodules negative for BRAF^V600E^ mutation on initial analysis.

BRAF^V600E^ mutation rate of the malignant nodules included in this study was 58.3% (7 of 12 nodules), which is lower than the reported 70–80% prevalence among Korean popluation [4], [16]. This may be explained by the inclusion of thyroid nodules which had initial negative BRAF^V600E^ mutation results. Our study included 49 thyroid nodule which had undergone two consecutive rounds of US-FNA and BRAF^V600E^ mutation analysis, all showing negative BRAF^V600E^ mutation on initial analysis. When putting US assessements of the 49 thyroid nodules in consideration, all 17 nodules with probably benign features had negative results on follow-up BRAF^V600E^ mutation analysis and finally confirmed as benign. Many recent reports show low malignancy rates among thyroid nodules diagnosed as non-diagnostic, benign or atypia on initial cytology showing no suspicious US features [21], [32]–[33], which may be further supported by the fact that this study included nodules with negative results on initial BRAF^V600E^ mutation analysis. In contrast, 7 of the 32 nodules with suspicious malignant US assessment were positive for BRAF^V600E^ mutation on follow-up analysis. Follow-up cytology results of the 7 nodules were benign (n = 1), indeterminate (n = 1), suspicious for malignancy (n = 4), and malignancy (n = 1). The Bethesda System for Reporting Thyroid Cytopathology recommends repeating US-FNA on thyroid nodules with non-diagnostic or atypia, and diagnostic lobectomy on those with indeterminate or suspicious for malignancy [21]. Similarly, the American Thyroid Association management guidelines recommend follow-up US-FNA on nodules diagnosed as non-diagnostic or atypia on cytology, and either lobectomy or total thyroidectomy on those diagnosed as indeterminate and suspicious for malignancy [15]. Follow-up US-FNA is also recommended on thyroid nodules showing suspicious US features which are diagnosed as benign on cytology [34]–[36]. When considering these recommendations, follow-up BRAF^V600E^ mutation analysis confidently lead surgeons towards deciding upon therapeutic surgery in 5 nodules with indeterminate and suspicious for malignancy on follow-up cytology. Also, false-negative results can occur in US-FNA [11], [37]. and follow-up BRAF^V600E^ mutation contributed in detecting malignancy in 1 nodule with false-negative cytology.

Diagnostic performances analysed in this study shows that when combining the follow-up BRAF^V600E^ mutation analysis results to follow-up US-FNA alone, sensitivity was significantly improved, 83.3% to 91.7%, with specificity 100% in all combinations. Among the 32 thyroid nodules with suspicious US features, 11 benign nodules had benign cytology on initial and follow-up US-FNA, along with negative results on the two consecutive BRAF^V600E^ mutation analyses. When considering the high specificity of BRAF^V600E^ mutation analysis, two consecutive negative results of BRAF^V600E^ mutation analysis along with negative cytology results may reassure clinicians in deciding upon follow-up rather than diagnostic lobectomy, reducing unnecessary diagnostic surgical procedures. But, among the 7 nodules showing positive results on follow-up BRAF^V600E^ mutation analysis, 6 were had indeterminate, suspicious for malignancy or malignant cytology results on follow-up US-FNA, presenting suspicious US features. These results alone without the addition of follow-up BRAF^V600E^ mutation analysis may be an indication for therapeutic surgery. But, US features in which surgeons rely upon in deciding surgery involves a significant amount of subjectivity produced by the operator, therefore, BRAF^V600E^ mutation analysis has its role in that it provides more objective information regarding the targeted nodule. Although follow-up BRAF^V600E^ mutation analysis may not be useful in nodules diagnosed as malignancy on follow-up US-FNA, it still has its strong points in choosing upon therapeutic methods among nodules with indeterminate or suspicious for malignancy results on follow-up US-FNA.

Differences in BRAF^V600E^ mutation results between US-FNA cytology and pathology specimen obtained with surgery have been reported in several studies [7], [38]. As in a study on detection of BRAF^V600E^ mutation in indeterminate thyroid nodules [38], the tumor cell contents of cytology specimen can limit the detection of BRAF^V600E^ mutation; even if the cancer tissues contain mutant cells, if the cytology specimen does not include enough mutant cells BRAF^V600E^ mutation analysis may show negative results. Recently, highly sensitive analysis methods compared to direct DNA sequencing such as pyrosequencing and dual priming oligonucleotide (DPO)- based multiplex polymerase chain reaction (PCR) have been introduced, showing abilities to detect less than 2–10% of mutant cells among the aspirated specimen [22], [39], but still, it is clear that obtaining enough mutant cells during US-FNA procedures is important in getting accurate results. The gap between the two consecutive BRAF^V600E^ mutation analysis of our study may be explained by the differences between the initial and follow-up cytology results of the 7 nodules; while initial cytology results were non-diagnostic, benign or atypia, follow-up cytology consists of suspicious for malignancy or malignancy in 5 of the 7 nodules. In contrast to the previously mentioned studies, although obtaining enough cells during aspiration is critical, BRAF^V600E^ mutation analysis has shown high sensitivity regardless of the analysis method, even in cytology specimen somewhat insufficient for accurate diagnosis [22], [40]–[41]. Even though BRAF^V600E^ mutation has its limitations, it has improved the selection of thyroid nodules for surgical treatment in our study; BRAF^V600E^ mutation was detected in the 2 nodules with benign and indeterminate follow-up cytology results in our study, further confirming its efficacy in the diagnosis of PTC.

There are several limitations to our study. First, a limited number of patients and thyroid nodules with initial and follow-up BRAF^V600E^ mutation were included in this retrospective study. As the indication or the cost-effectiveness of follow-up BRAF^V600E^ mutation analysis has not yet been proven, few patients had undergone follow-up analysis, and selection bias may have existed. Further studies in a large series is anticipated. Second, 14 radiologists with various experiences in thyroid imaging were involved in US assessment and US-FNA procedures. Observer variability among radiologists may have affected US assessments and cytology results of thyroid nodules. Similarly, 5 cytopathologists were involved in cytology slide interpretation, and interobserver variability among cytopathologists may have affected the US-FNA cytology results. Last, 2 of the 13 malignant nodules were diagnosed based on cytology only, and the 17 benign nodules were considered benign based on cytology results and US follow-up examinations. False-positive and false-negative results can occur in US-FNA, reported false-positive rates are 0.2–5.7% [42]–[43], false-negative rates 1.9–5.8% [34], [44], which may have had effect on our results.

In conclusion, follow-up BRAF^V600E^ mutation analysis may be helpful in the diagnosis of selected thyroid nodules negative for BRAF^V600E^ mutation on initial analysis, which are assessed as suspicious malignant on US, diagnosed as non-diagnostic, benign or atypia on follow-up US-FNA.
